# Data set of *in-silico* analysis and 3D modelling of boiling stable stress-responsive protein from drought tolerant wheat

**DOI:** 10.1016/j.dib.2019.104657

**Published:** 2019-10-30

**Authors:** Arun Dev Sharma, Gurmeen Rakhra, Dhiraj Vyas

**Affiliations:** a PG Department of Biotechnology, Lyallpur Khalsa College, G.T. Road, Jalandhar, 144001, Punjab, India; b Biodiversity and Applied Botany Division Indian Institute of Integrative Medicine (CSIR), Canal Road, Jammu, 180001, India

**Keywords:** Amphipathic α helices, Boiling soluble proteins, Drought stress, In-silico analysis, Wheat

## Abstract

Boiling stable proteins are widespread, evolutionary conserved proteins from several kingdoms including plants, fungi and bacteria. Accumulation evidences in response to dehydration, suggest a wide spread adaptation and an evolutionary role of these protein families to protect cellular structures from water loss effects in a wide range of water potentials. Boiling stable proteins, although represents just 0.1% of total plant proteins, resist coagulation upon boiling and believed to be involved in water stress adaptation in plants. The present data profiles *in-silico* analysis of cloned boiling stable protein encoding gene wBsSRP from drought tolerant cultivar of wheat. The data presented here was of a gene isolated from total RNA/mRNA samples of wheat variety PBW 175 subjected to drought stress. The gene is available with EMBL data repository with accession number LN832556.

**Table:** Specifications Table

Subject	Biology
Specific subject area	*In-silico* analysis of drought responsive gene
Type of data	Data tables and figures in word files
How data were acquired	Gene was isolated by –RT-PCR and *in-silico* analysis was done by using BLAST, CLUSTAL W, I-TASSER, VADAR, PDBsum and PROFUNC, tools
Data format	Raw and refined data
Experimental factors	Total RNA extracted from the drought stressed seedlings of drought tolerant cv. PBW 175. cDNA synthesis and PCR amplification using specific primers. Cloned in TA cloning vector pTZ57R/T
Experimental features	*In-silico* analysis and characterization of cloned gene
Data source location	Lyallpur Khalsa College, Jalandhar
Data accessibility	Cloned gene is available in EMBL database with accession number LN832556
Related research article	G. Rakhra, T. Kaur, D. Vyas, A.D.Sharma, J. Singh, G. Ram, Molecular cloning, characterization, heterologous expression and in- silico analysis of disordered boiling soluble stress-responsive wBsSRP protein from drought tolerant wheat cv.PBW 175. Plant Physiology Biochemistry, 112 (2017). 29-44

**Table:** 

**Value of the Data** • The data profiles *in-silico* and 3D modelling of boiling stable protein encoding gene • Data can be used to provide in-depth knowledge that boiling stable proteins might play an important role in the protection of plants under water, salt, ionic, cold or heat stress conditions. • This data can provides new insights to studies using diverse cultivars under control and drought, to see boiling soluble protein both at pre-flowering and post-flowering stages of the plant development in order to validate its role as a potential drought stress related marker.

## 1 Data

**Fig. 1:**
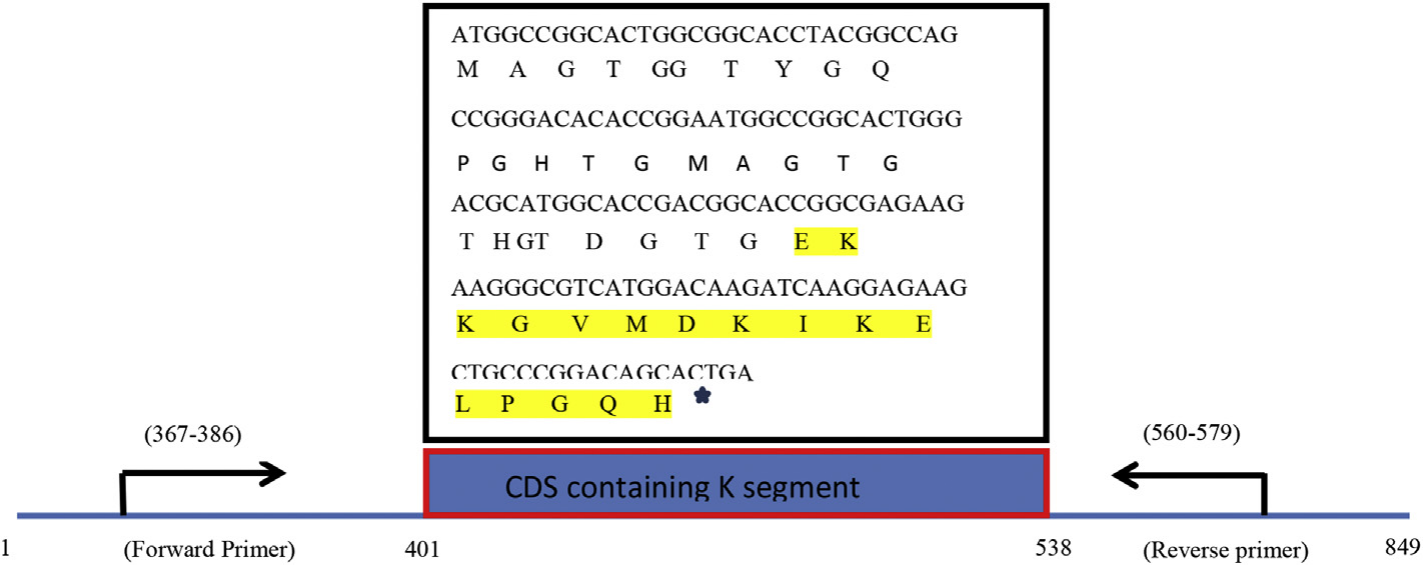
Schematic representation of the 849 bp WZY2 gene (LEA II family gene; accession no: EU395844) showing the positions of forward and reverse primers as well as the CDS containing K segment. The amino acid residues marked in yellow shows the peculiar K segment region in WZY2 gene. The * sign shows the termination codon of the CDS region.

**Fig. 2:**
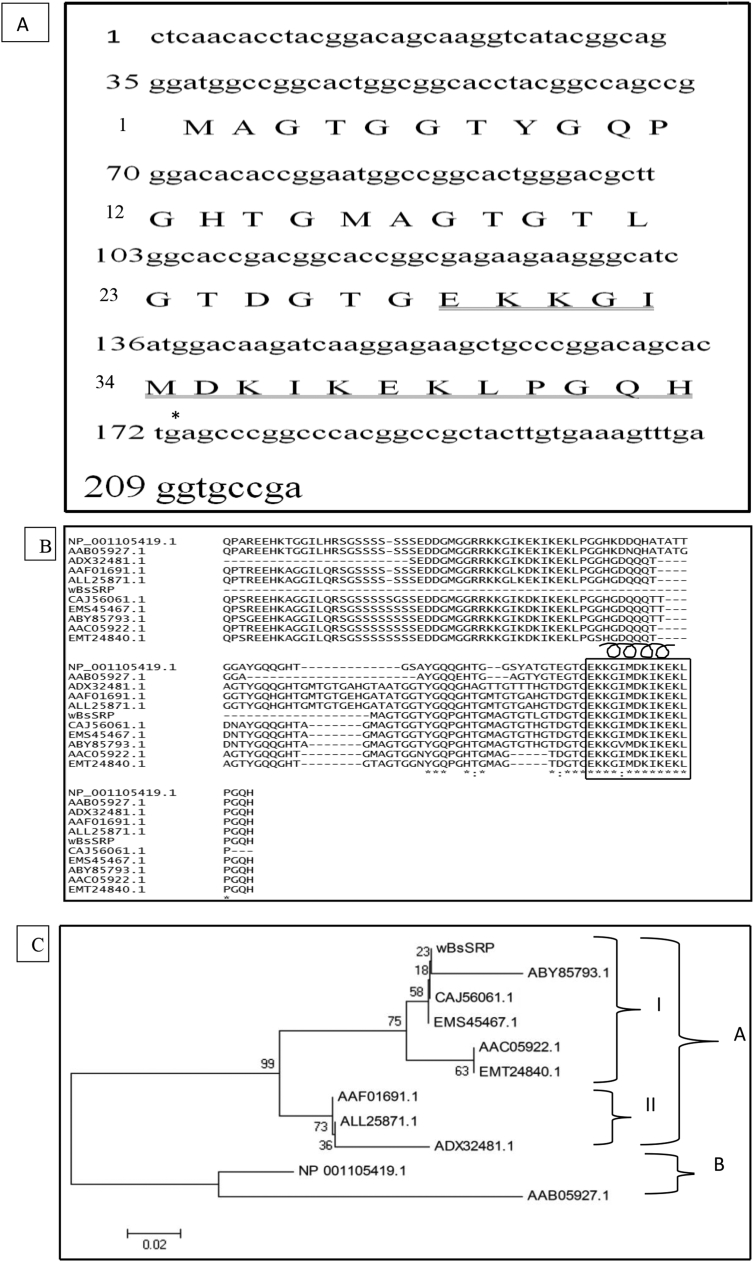
(A) Nucleotide sequence of the wheat *wBsSRP* gene and the deduced protein sequence. Amino acids are printed in upper case letters while the nucleotide sequence is shown in lower case letters. Amino acid residues corresponding to the K-segment forming an amphipathic α helix are marked with a double line. The nucleotides and amino acids are numbered on the left hand side. This sequence has been deposited in EMBL GenBank databases under the accession number LN832556. An asterisk indicates the termination of protein. (B) Comparison of deduced amino acid sequence of wBsSRP with homologues dehydrinproteins from other plant species. Multiple sequence alignments were performed with Clustal-W (http://www.ebi.ac.uk/Tools/clustalw/index.html). Conserved amino acid residues corresponding to the lysine-rich K segment present in dehydrins of different plant species are boxed. The part of the K-segment in wBsSRP forming an amphipathic α helix is shown by a spiral. Accession numbers of the different dehydrin proteins from various plant species are listed in Table 1B. (C) Phylogenetic tree of wBsSRPwas constructed based upon aligned protein sequences from various plants using Bootstrap Neighbour Joining method by MEGA 4 tool. Letter code A and B denote two major groups while I and II denote the subgroups. Accession numbers belonging to different dehydrin proteins from various plant species are listed in Table 1B.

**Fig. 3:**
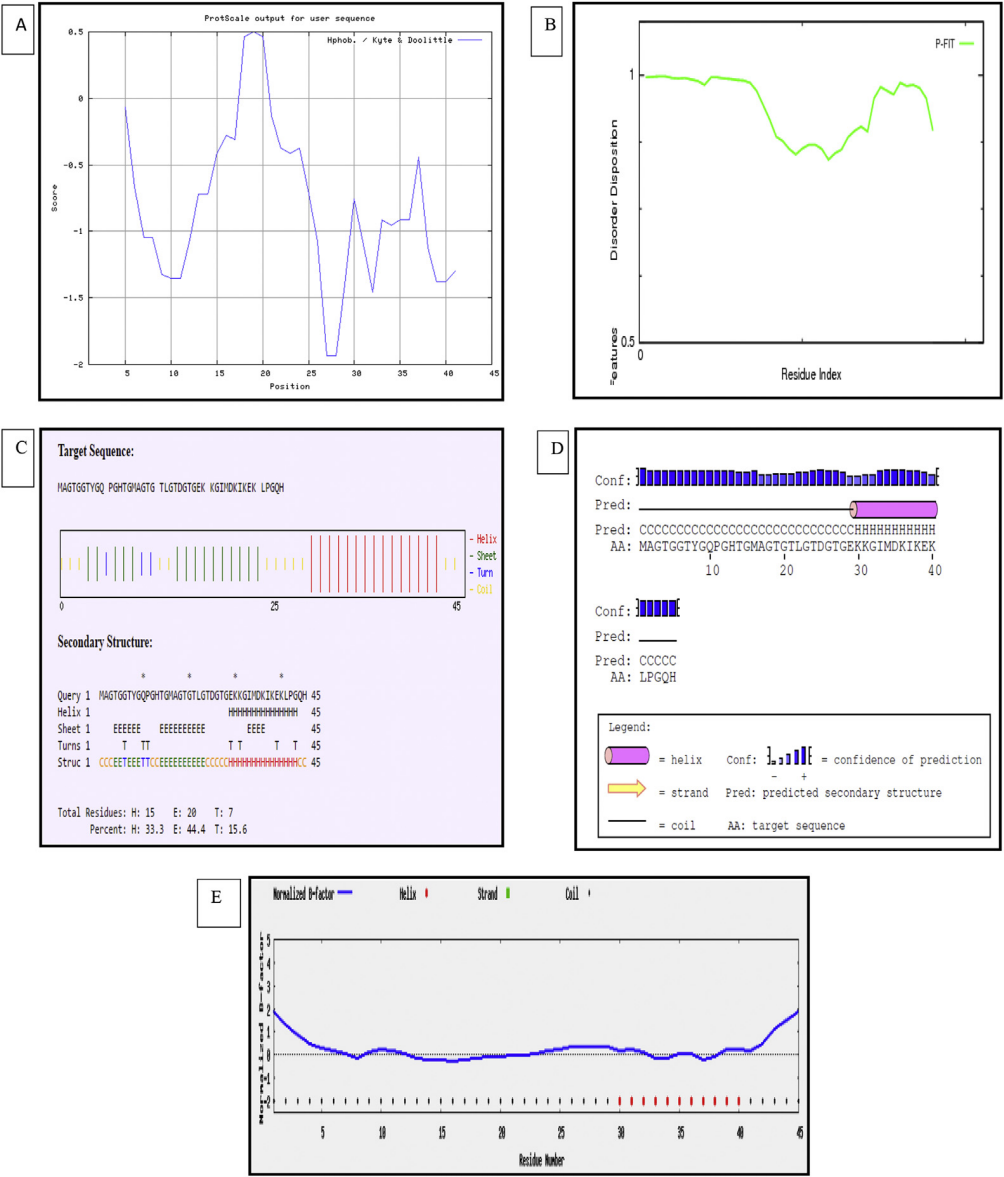
Hydropathy analysis (A), PONDR-fit (B), Chou Fasman (C), PSIPRED (D) and predicted normalized B- Factor (E) of wBsSRP sequence.

**Fig. 4:**
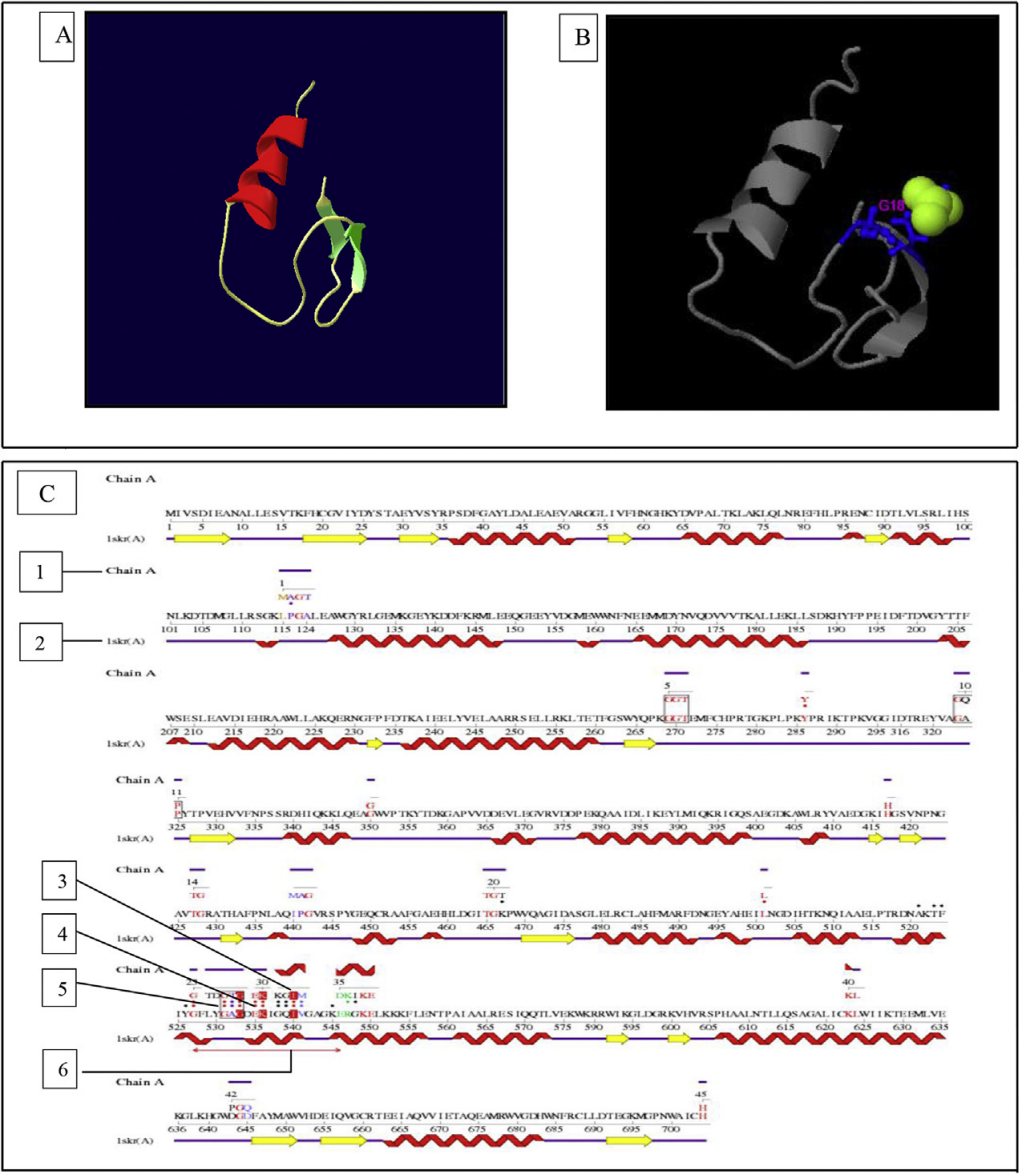
(A) Ribbon display of three dimensional (3D structure) of wBsSRP as predicted by I-TASSER server, using the crystal structure of PDB code1zvoCas template. Helices are in red; sheets are in green (colour figure online). (B)The best identified ligand binding site as predicted by I-TASSER web server is depicted in the ribbon model. The ligand binding site is displayed in the figure with the predicted binding ligand BCT shown in green yellow sphere while the binding residues G18 are shown in blue ball and stick. (C) Sequence alignment of the DNA binding template predicted by ProFunc tool server. The sequence alignment has been driven by the residues equivalenced by the template match. The sequences of the query and target proteins are aligned using the matched residues from the template search, together with any equivalenced residues within 10 Å of the template centre, to drive the alignment. Show the amino acid sequence, residue numbers and secondary structure “wiring diagram” of the query protein. The wiring diagram schematically illustrates the protein's helices as the red jagged elements and its beta strands as the yellow arrows. The sequence itself is coloured according to the residue similarity to the aligned residues in the target protein. Show the amino acid sequence residue numbers and secondary structure “wiring diagram” of the target protein. The wiring diagram schematically illustrates the protein's helices as the red jagged elements, its beta strands as the yellow arrows, and its coil regions as purple lines. The sequence itself is coloured according to the residue similarity to the aligned residues in the target protein. Correspond to the template residues: the residues highlighted in red correspond to the template residues and the equivalent residues in the other structure that they matched. Equivalenced residues: the dots identify which residues in each sequence lie within 10 Å of the template centre and hence show which were used to drive the alignment. Boxed regions: the boxed regions of the alignment represent segments where the sequence identity of the two sequences exceeds 35%; that is, regions of reasonably significant sequence similarity. Fittable regions: the red line segment identifies the structurally “fittable” and conserved functional region, common to these two proteins in the alignment. This corresponds to the segment from both proteins whose C-alpha coordinates can be structurally superposed with an r.m.s.d. of less than 3.0 Å.

**Fig. 5:**
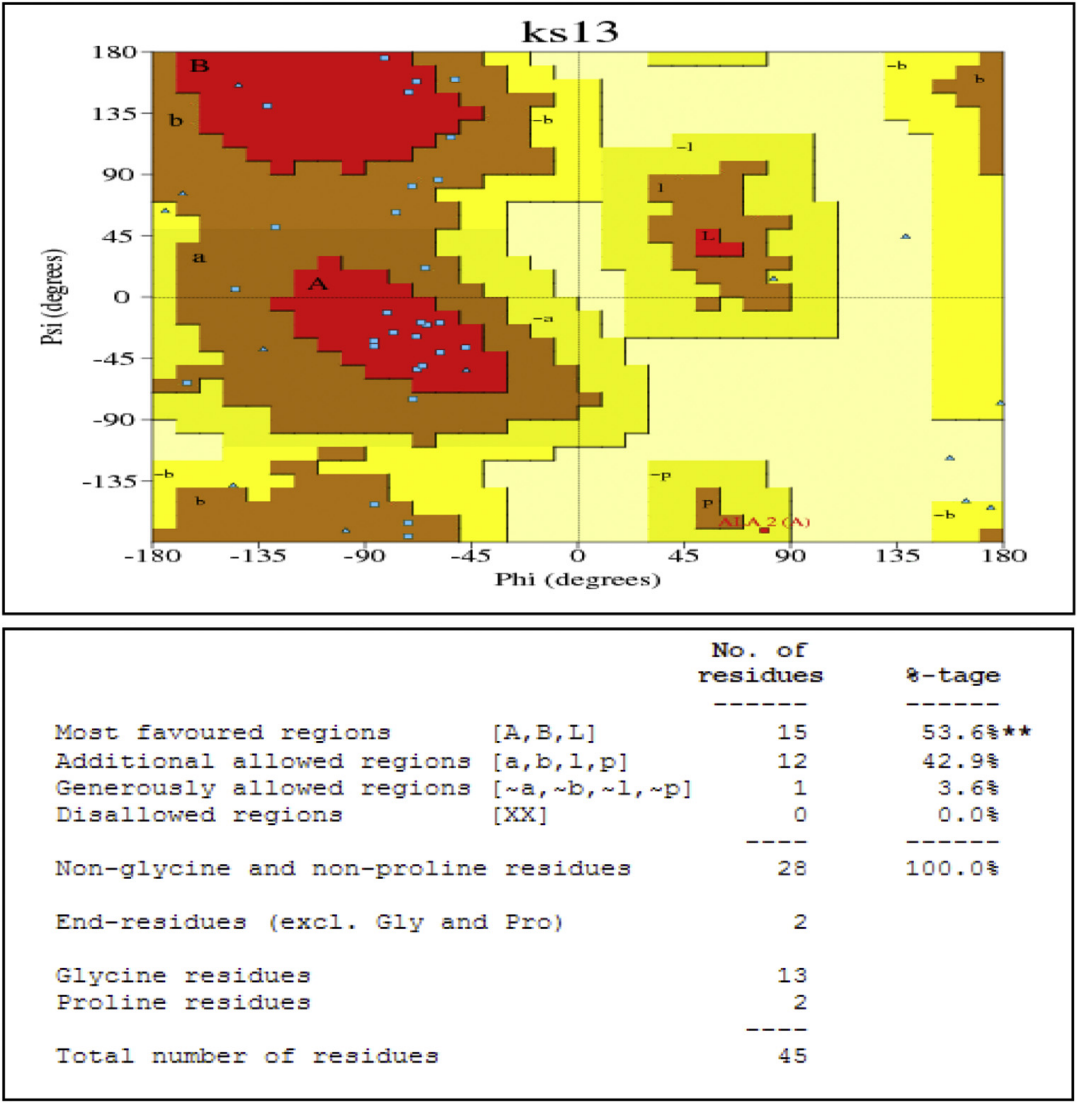
Ramachandran plot analysis. The plot calculations were computed by PROCHECK server. The red regions in the graph indicate the most allowed regions [A, B, L], additional allowed regions [a, b, l, p] are indicated as brown, generously allowed regions [∼a,∼b,∼l,∼p] are indicated as green and yellow shades.

**Fig. 6:**
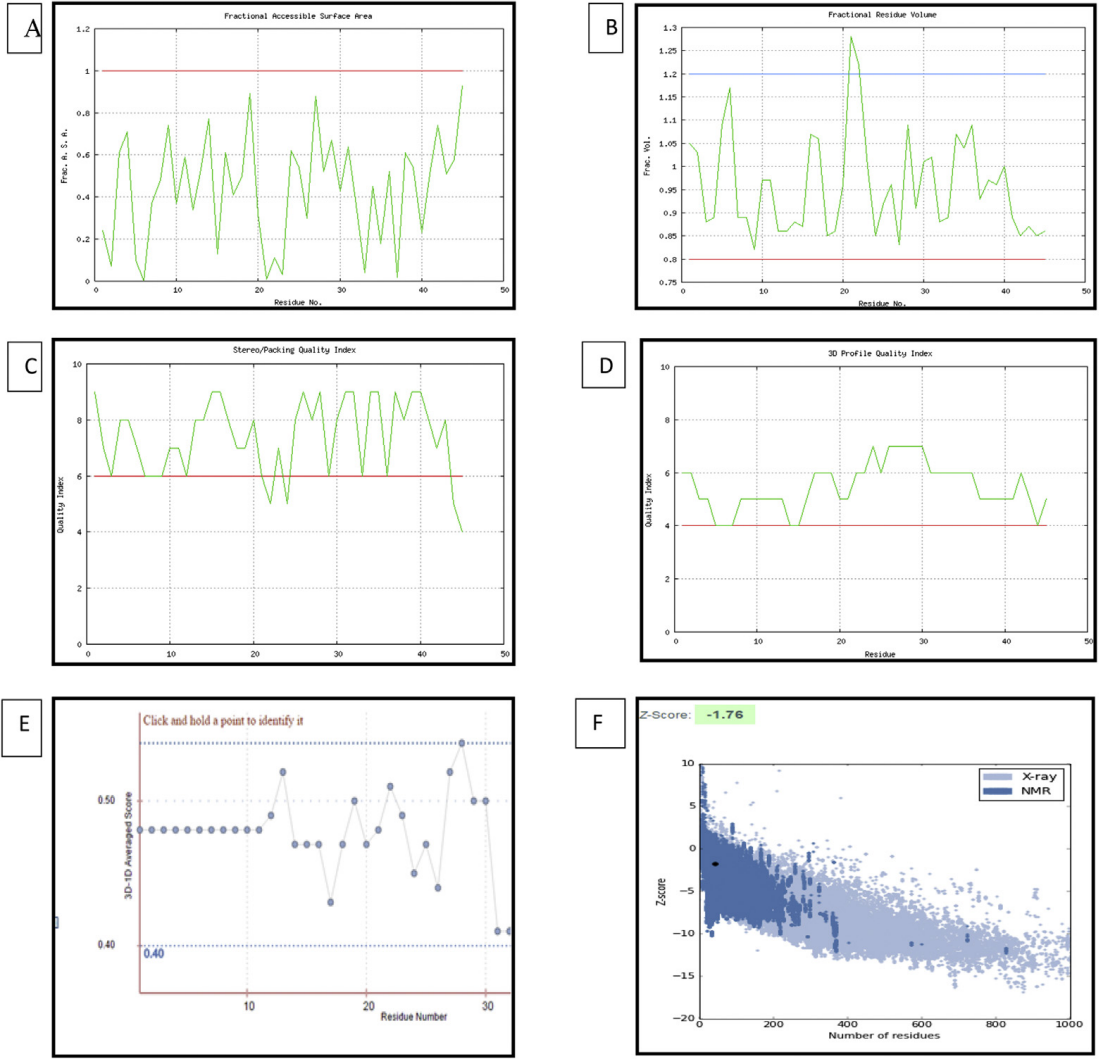
Structural verification by VADAR (A,B,C,D), Verify 3D (E), PROSA (F).

**Fig. 7:**
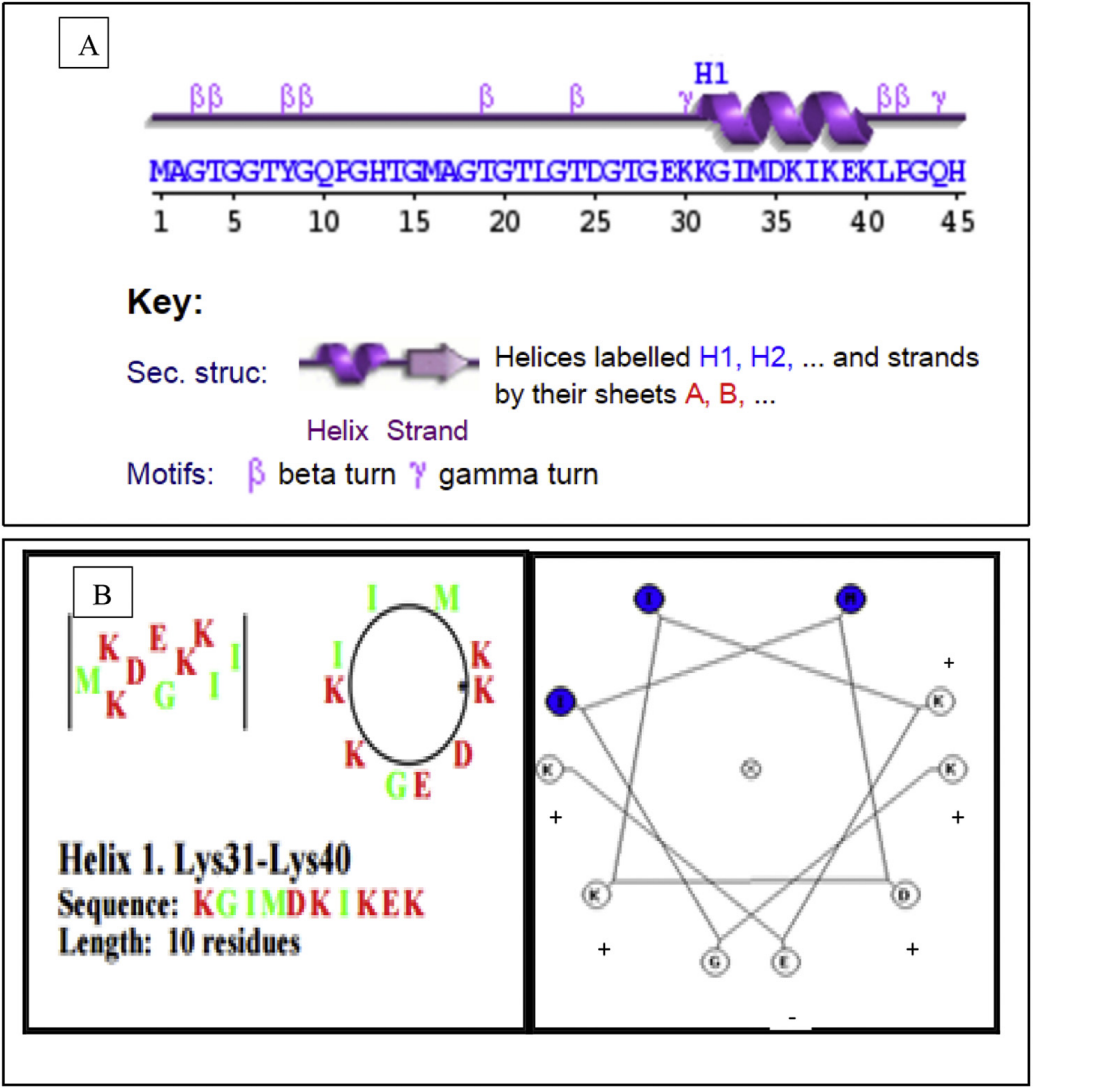
(A) Structural motif analysis of wBsSRP generated by PDBsum server (Colour figure online) (B) Helical wheel diagram of the lysine rich K- segment forming an amphipathic α helix. The hydrophobic residues are marked in green and blue while hydrophilic residues are marked in red and empty circles (B). (+) sign on empty circles correspond to positive residues while (−) sign correspond to negative residues.

**Fig. 8:**
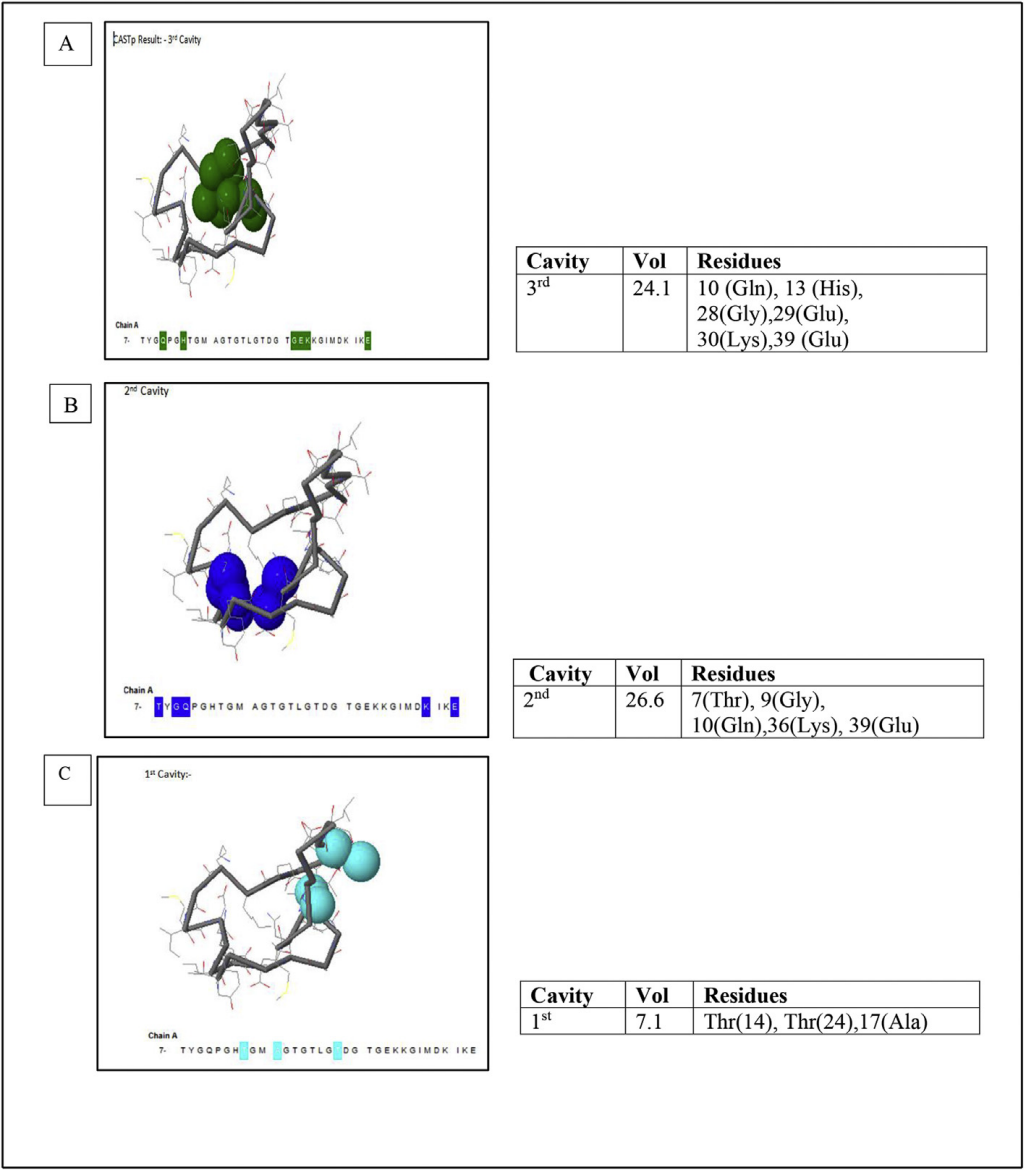
Major binding clefts/cavities in wBsSRP protein 3D structure and wBsSRP protein sequence showing the position of active site residues among various cavities as identified by CASTp binder site prediction tool. Colour coding scheme: cavity 1: light blue, cavity 2: blue, cavity 3: green. Boxed residues are the active site residues among various cavities.

This dataset represents *in-silico* and 3D modelling of a drought stress responsive gene encoding a boiling stable protein. One microliter of cDNA prepared from drought stressed leaves of tolerant cultivar of wheat PBW 175 was used as a template for using RT-PCR amplification of CDS (protein coding sequence) encoding hydrophilic protein having K-segment with a pair of gene-specific primers (*WZY2* gene, LEA II family gene; accession no: EU395844) (Fig. 1). This stress related gene was submitted to EMBL GenBank and was designated as wBsSRP (wheat boiling soluble stress responsive protein; accession number LN832556). An ORF encoding 45 amino acid long protein sequence was retrieved and subjected to BLAST-P and BLAST-N analysis (Table 1, Fig. 2A). Multiple amino acid sequence alignment (Fig. 2B), indicated a typical conserved signature sequence. The phylogeny data analysis tree construction depicted existence of two major groups namely A and B (Fig. 2C). Physio-chemical properties of the protein sequence were computed by Protparam tool (Table 2). Glycine content was more as compared to other amino acids (Supplementary Fig 1). Hydropathy plot data using Kyte Doolite scale, is shown in Fig. 3A and Supplementary Fig 2. Structural disorder by *in-silico* predict done by PONDR-fit (Fig. 3B and Supplementary Fig 3). The secondary structure prediction data by Chou Fasman (Fig. 3C). PSIPRED also validated the presence of helix in the protein sequence (Fig. 3D). Thermal mobility of residues is defined by B- factor profile (BFP) (Fig. 3E). I-TASSER was used for 3D modelling (Table 3A, Table 3B). Threading based modelling of wBsSRP protein by I-TASSER server predicted ligand binding sites (Fig. 4B). Functional prediction was carried out using Profunc tool (Fig. 4C). Validity and quality of model was checked by Ramachandran plot (Fig. 5). And VADAR, and PROSA (Fig. 6 and Supplementary Fig 4) which indicated a good three dimensional model. PDBsum server used for structural motif assessment (Fig. 7A). Helical wheel diagram of the K- segment in wBsSRP protein predicted helix was amphipathic containing hydrophobic (marked in green and blue) on one side and hydrophilic residues (marked in red and empty circles) on the other side of the helix (Fig. 7B and Supplementary Fig 5). Active sites were predicted by CAST P tool (Fig. 8).

**Table 1:** Homology search of the *wBsSRP* gene (A) and protein sequence (B) for deducing similarity with available sequences in databases using BLAST N and BLAST P at NCBI database (www.ncbi.nlm.nih.gov)**.**

A						
Name of the protein	Max score	Total score	Query cover	E value	Identity	Accession number
*Triticum aestivum* cultivar Zhengyin 1 dehydrin (wzy2) gene, complete cds	383	383	98%	8e-103	99%	KF112871.1
*Triticum turgidum* subsp. *durum* partial mRNA for dehydrin 3 (DHN15.3 gene)	285	285	74%	2e-73	99%	AM180931.1
*Hordeum vulgare* dehydrin (Dhn7) mRNA, complete cds	244	244	79%	4e-61	92%	AF181457.1
*Hordeum vulgare* subsp. *vulgare* cultivar Morex dehydrin 7 (Dhn7) gene, complete cds	239	239	79%	2e-59	92%	KC963090.1
*Hordeum vulgare* subsp. *spontaneum* voucher NPGS PI 531957 dehydrin 7 (Dhn7) gene, complete cds	239	239	79%	2e-59	92%	AY895929.1
*Secale cereale* cultivar Lo152 Dhn3 gene, exon 2 and partial cds	224	224	79%	5e-55	90%	HQ730771.1
*Lophopyrum elongatum* dehydrin-/LEA group 2-like protein (ESI18-3) mRNA, complete cds	196	196	97%	1e-46	85%	AF031248.1
*T.durum Desf. (Siliana)* Dehydrin mRNA, clone pTd16	187	187	97%	7e-44	84%	X78429.1
*Panicum miliaceum* dehydrin mRNA, complete cds	134	134	63%	9e-28	84%	KT438253.1
*Zea mays* dehydrin 1 (dhn1), mRNA	122	122	61%	2e-24	84%	NM_001111949.1
B						
Name of the protein	Max score	Total score	Query cover	E value	Identity	Accession number
Dehydrin DHN3 [*Triticum urartu*]	85.9	85.9	100%	3e-20	98%	EMS45467.1
Dehydrin [*Hordeum vulgare* subsp. *vulgare*]	72.0	72.0	93%	6e-15	90%	AAF01691.1
Dehydrin3 [*Hordeum vulgare* subsp. *spontaneum*]	72.0	72.0	93%	7e-15	90%	ALL25871.1
dehydrin-/LEA group 2-like protein [*Thinopyrum elongatum*]	71.6	71.6	100%	9e-15	87%	AAC05922.1
dehydrin WZY2 [*Triticum aestivum*]	64.3	64.3	100%	5e-12	96%	ABY85793.1
dehydrin 3, partial [*Triticum turgidum* subsp. *durum*]	59.3	59.3	93%	5e-10	98%	CAJ56061.1
dehydrin DHN1 [*Zea mays*]	58.2	58.2	97%	2e-09	67%	NP_001105419.1
Dehydrin DHN3 [*Aegilops tauschii*]	57.4	57.4	80%	3e-09	86%	EMT24840.1
dehydrin [*Sorghum bicolor*]	55.8	55.8	95%	4e-09	74%	AAB05927.1
Dhn3 [*Secale cereale*]	52.8	52.8	93%	5e-08	54%	ADX32481.1

**Table 2:** Physicochemical properties of wBsSRP.

Number of amino acids	45
pI	8.14
Molecular weight	4473
Total number of negatively charged residues (Asp + Glu)	4
Total number of positively charged residues (Arg + Lys)	5
Ext.coefficient	1490
Abs 0.1% (=1g/l)	0.333
Estimated half- life (N terminal of the sequence considered is M (Met)	30 hours (mammalian reticulocytes, in vitro), >20 hours (yeast, in vivo), >10 hours (*Escherichia Coli*, in vivo).
Instability index (II)	−8.86
Aliphatic Index	39.11
Grand average of hydropathicity (GRAVY)	−0.791

**Table 3A:** List of top ten templates used by I- TASSER for 3D structure prediction of wBsSRP.

S.No	PDB hits
1	1zvoC
2	2kfeA
3	2kk7A
4	2kfeA
5	1ddzA
6	3u1cA
7	3itcA
8	2rb6A
9	2hgqE
10	2i9oA

**Table 3B:** Model evaluation data for the predicted structure of wBsSRP protein.

Model	C-score	Exp. TM Score	Exp. RMSD	No. of decoys	Cluster density
1	−2.59	0.41 ± 0.14	7.7 ± 4.3	4300	0.0994
2	−2.99			2623	0.0667
3	−3.73			1220	0.0318
4	−2.98			2850	0.0669
5	−5.00			110	0.0076

## 2 Experimental design, materials, and methods

### 2.1 Plant material and growth conditions

The seeds of drought to tolerant cultivar of *Triticum aestivum* L. cv. PBW 175 [1] was surface sterilized, imbibed for 6 h and germinated for three days. Drought stress was imposed to 3- day old seedlings for 48 h by withholding water supply.

### 2.2 PCR amplification and cloning of wBsSRP gene

Using Nucleospin RNA plant isolation kit (Macherey Nagel, Duren, Germany), total RNA was extracted from the drought stressed seedlings of drought tolerant cv. PBW 175 using instructions. One μg of RNA sample was reverse transcribed using “Transcriptor High Fidelity cDNA Synthesis Kit” (Roche Diagnostics, Mannheim, Germany) with oligodT as a primer. One microliter of cDNA was used as a template for PCR amplification of CDS (protein coding sequence) encoding hydrophilic protein having K-segment with a pair of gene-specific primers (*WZY2* gene, LEA II family gene; accession no: EU395844) (Fig. 1) following Rakhra et a (2017) [2]. The gene was successfully accessioned in EMBL GenBank with accession number LN832556. *wBsSRP* gene was cloned TA cloning vector pTZ57R/T using “InsTAclone” TM (Thermo Fisher Scientific, Waltham, Massachusetts, USA).

### 2.3 Sequence analysis of wBsSRP

ORF Finder tool at NCBI (www.ncbi.nlm.nih.gov) to identify the coding regions. The *wBsSRP* gene and protein sequence was subjected to homology search using BLAST at NCBI database for deducing similarity with available sequences in databases (www.ncbi.nlm.nih.gov). Conserved region analysis among various protein homologues were carried out using CLUSTAL-W tool (http://www.ebi.ac.uk/Tools/msa/clustalw2/). Phylogenetic tree was constructed based on aligned protein sequences from various plants using Bootstrap Neighbour Joining method by MEGA 4 tool [3]. Physicochemical properties was calculated by protparam tool at expasy (www.expasy.org). Chou Fasman (www.biogem.org/tool/chou-fasman/) and PSIPRED (http://bioinf.cs.ucl.ac.uk/psipred/) tools were used for secondary structure prediction from the amino acid sequence. Hydropathy analysis was carried out using Protscale at Expasy with following parameters: scale: Hphob./Kyte & Doolittle; window size: 9; weight variation model: linear. PONDR-fit tool was used to identify intrinsically disorder nature of protein (http://www.pondr.com/) using VLXT predictor.

### 2.4 Molecular modelling (3-D) and evaluation of wBsSRP protein

The three dimensional structure of wBsSRP protein was predicted by iterative threading assembly refinement algorithm (I- TASSER) Standalone package (Version 1.1) [4].

### 2.5 Validations, structural and functional analysis

Structural analysis, validations were done using VADAR (http://redpoll.pharmacy.ualberta.ca/vadar), using following Programme options: Vandel Wall raii Sharke, Standard Voronoi procedure for value calculation. PROSA (http://prosa.services.came.sbg.ac.at/prosa.php), Phi/Psi Ramachandran plot (www.ebi.ac.uk/pdbsum). PDB sum was used to find out structural motifs. ProFunc server of EMBL-EBI was used to identify the likely biochemical function. Helical wheel prediction was carried out using Pepwheel tool using following parameters: number of steps:18, turns :5 and output format: PNG (http://www.bioinformatics.nl/cgi-bin/emboss/pepwheel). Helixator was also used to find out amphipathic TMCs (http://www.tcdb.org/progs/helical_wheel.php).

### 2.6 Catalytic active site prediction

CASTp (Computed Atlas of Surface Topography of proteins) was used to find out catalytic sites (http://sts.bioengr.uic.edu/castp/calculation.php).
