# Supplementary material for: Data set of in-silico analysis and 3D modelling of boiling stable stress-responsive protein from drought tolerant wheat
Source: Data Brief. 2019 Oct 30;27:104657. doi: 10.1016/j.dib.2019.104657 (PMC6849113; doi:10.1016/j.dib.2019.104657)
Supplement: Multimedia component 1 [file mmc1.docx]

**Supplementary Fig 1**

[ProtParam](https://web.expasy.org/protparam) [Home](https://web.expasy.org/protparam) | [**Contact**](https://web.expasy.org/contact)

**ProtParam**

**User-provided sequence:**

10 20 30 40

MAGTGGTYGQ PGHTGMAGTG TLGTDGTGEK KGIMDKIKEK LPGQH

[References](https://web.expasy.org/protparam/protpar-ref.html) and [documentation](https://web.expasy.org/protparam/protparam-doc.html) are available.

# Number of amino acids: 45

**Molecular weight:** 4473.03

# Theoretical pI: 8.14

**Amino acid composition:**


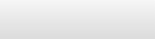


CSV format

| Ala | (A) | 2 | | 4.4% |
| --- | --- | --- | --- | --- |
| Arg | (R) | 0 | | 0.0% |
| Asn | (N) | 0 | | 0.0% |
| Asp | (D) | 2 | | 4.4% |
| Cys | (C) | 0 | | 0.0% |
| Gln | (Q) | 2 | | 4.4% |
| Glu | (E) | 2 | | 4.4% |
| Gly | (G) | 13 | | 28.9% |
| His | (H) | 2 | | 4.4% |
| Ile | (I) | 2 | | 4.4% |
| Leu | (L) | 2 | | 4.4% |
| Lys | (K) | 5 | | 11.1% |
| Met | (M) | 3 | | 6.7% |
| Phe | (F) | 0 | | 0.0% |
| Pro | (P) | 2 | | 4.4% |
| Ser | (S) | 0 | | 0.0% |
| Thr | (T) | 7 | | 15.6% |
| Trp | (W) | 0 | | 0.0% |
| Tyr | (Y) | 1 | | 2.2% |
| Val | (V) | 0 | | 0.0% |
| Pyl | (O) | 0 | | 0.0% |
| Sec | (U) | 0 | | 0.0% |
| (B) | 0 | | 0.0% | |
| (Z) | 0 | | 0.0% | |
| (X) | 0 | | 0.0% | |

**Total number of negatively charged residues (Asp + Glu):** 4

**Total number of positively charged residues (Arg + Lys):** 5

**Atomic composition:**

| Carbon | C | 188 |
| --- | --- | --- |
| Hydrogen | H | 308 |
| Nitrogen | N | 56 |
| Oxygen | O | 64 |
| Sulfur | S | 3 |

**Formula:** C188H308N56O64S3

# Total number of atoms: 619

**Extinction coefficients:**

This protein does not contain any Trp residues. Experience shows that

this could result in more than 10% error in the computed extinction coefficient.

Extinction coefficients are in units of M-1 cm-1, at 280 nm measured in water. Ext. coefficient 1490

Abs 0.1% (=1 g/l) 0.333

# Estimated half-life:

The N-terminal of the sequence considered is M (Met).

The estimated half-life is: 30 hours (mammalian reticulocytes, in vitro).

>20 hours (yeast, in vivo).

>10 hours (Escherichia coli, in vivo).

# Instability index:

The instability index (II) is computed to be -8.86 This classifies the protein as stable.

**Aliphatic index:** 39.11

# Grand average of hydropathicity (GRAVY): -0.791

[SIB Swiss Institute of Bioinformatics](https://sib.swiss/) | [Disclaimer](https://www.expasy.org/disclaimer.html)
