# Supplementary material for: Data set of in-silico analysis and 3D modelling of boiling stable stress-responsive protein from drought tolerant wheat
Source: Data Brief. 2019 Oct 30;27:104657. doi: 10.1016/j.dib.2019.104657 (PMC6849113; doi:10.1016/j.dib.2019.104657)
Supplement: Multimedia component 2 [file mmc2.docx]

**Supplementary Fig 2**

[ProtScale](https://web.expasy.org/protscale) [Home](https://web.expasy.org/protscale) | [**Contact**](https://web.expasy.org/contact)

**ProtScale**

**User-provided sequence:**

10 20 30 40

MAGTGGTYGQ PGHTGMAGTG TLGTDGTGEK KGIMDKIKEK LPGQH

SEQUENCE LENGTH: 45

Using the scale [**Hphob. / Kyte & Doolittle**](https://web.expasy.org/protscale/pscale/Hphob.Doolittle.html), the individual values for the 20 amino acids are:

| Ala: 1.800 | Arg: -4.500 | Asn: -3.500 | Asp: -3.500 | Cys: 2.500 | Gln: -3.500 |
| --- | --- | --- | --- | --- | --- |
| Glu: -3.500 | Gly: -0.400 | His: -3.200 | Ile: 4.500 | Leu: 3.800 | Lys: -3.900 |
| Met: 1.900 | Phe: 2.800 | Pro: -1.600 | Ser: -0.800 | Thr: -0.700 | Trp: -0.900 |

Tyr: -1.300 Val: 4.200 : -3.500 : -3.500 : -0.490

Weights for window positions 1,..,9, using **linear weight variation model**:

| 1 | 2 | 3 | 4 5 6 | 7 | 8 | 9 |
| --- | --- | --- | --- | --- | --- | --- |
| 1.00 | 1.00 | 1.00 | 1.00 1.00 1.00 | 1.00 | 1.00 | 1.00 |
| edge |  |  | center |  |  | edge |


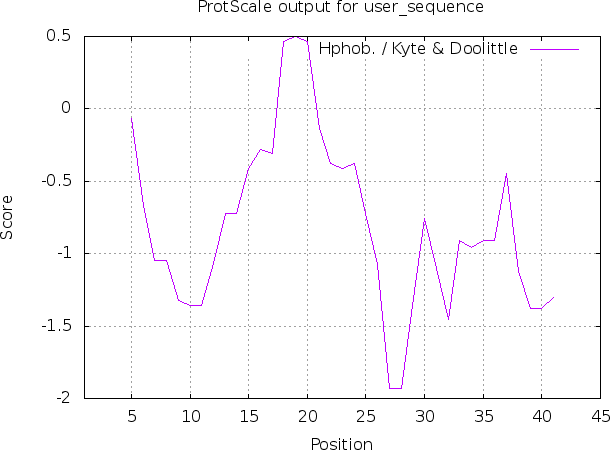


The results of your ProtScale query are available in the following formats: [Image in GIF-format](https://web.expasy.org/tmp/pscale147318.gif)

[Image in Postscript-format](https://web.expasy.org/tmp/pscale147318.ps) [Numerical format (verbose)](https://web.expasy.org/tmp/scores147318.txt)

[Numerical format (minimal, to be exported into an external application)](https://web.expasy.org/tmp/scoresmin147318.txt)

[SIB Swiss Institute of Bioinformatics](https://sib.swiss/) | [Disclaimer](https://www.expasy.org/disclaimer.html)
