# Supplementary material for: Data set of in-silico analysis and 3D modelling of boiling stable stress-responsive protein from drought tolerant wheat
Source: Data Brief. 2019 Oct 30;27:104657. doi: 10.1016/j.dib.2019.104657 (PMC6849113; doi:10.1016/j.dib.2019.104657)
Supplement: Multimedia component 3 [file mmc3.docx]

**Supplementary Fig 3**


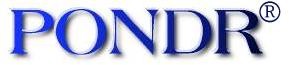


**Predictor of Natural Disordered Regions**

[PONDR® tutorial](http://www.pondr.com/pondr-tut.html)


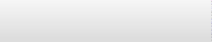

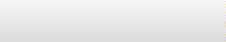


DEPP prediction

New Prediction


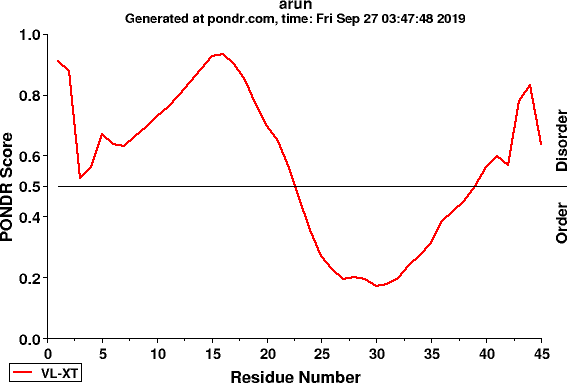


Note: Click on the picture to zoom in.

PONDR Protein Disorder Predictor

Developed by P. Romero, X. Li, A.K. Dunker,Z. Obradovic, E. Garner.

VL3 Predictor

Developed by P. Radivojac and A.K. Dunker

DEPP Predictor Developed by P. Radivojac

VSL2 Predictor Developped by K. Peng and Z. Obradovic

================================VLXT NNP STATISTICS================================

Predicted residues: 45 Number Disordered Regions: 2

Number residues disordered: 28 Longest Disordered Region: 22 Overall percent disordered: 62.22 Average Prediction Score: 0.5658 Predicted disorder segment [1]-[22] Average Strength= 0.7483 Predicted disorder segment [40]-[45] Average Strength= 0.6624

================================PREDICTOR OUTPUT================================

"D" = Disordered " " = Ordered

================================================================================

1 MAGTGGTYGQ PGHTGMAGTG TLGTDGTGEK KGIMDKIKEK LPGQH VLXT DDDDDDDDDD DDDDDDDDDD DD D DDDDD
