# Supplementary material for: Data set of in-silico analysis and 3D modelling of boiling stable stress-responsive protein from drought tolerant wheat
Source: Data Brief. 2019 Oct 30;27:104657. doi: 10.1016/j.dib.2019.104657 (PMC6849113; doi:10.1016/j.dib.2019.104657)
Supplement: Multimedia component 4 [file mmc4.docx]

**Supplementary Fig 4**

********************************************************************************

- Package...: VADAR (c)
- Version...: 1.5, December 2007
- Location..: University of Alberta
- Protein Engineering Network of
- Centres of Excellence
- Input.....: /var/www/html/vadar/public_html/temp/1569487517/1569487517.txt_m0

* Date......: Thu Sep 26 08:45:18 2019

********************************************************************************

**********************************

* VADAR STATS *

**********************************

** Using atomic radii from Shrake **

(The expected values represent those numbers which would be expected for highly refined Xray and NMR protein structures.

See the help pages for more information on expected values.)

|------------------------------|---------------------|---------------------|

| Statistic | Observed | Expected |

|------------------------------|---------------------|---------------------|

| \| | # | Helix | \| | 10 | ( | 22%) | \| | - \| |
| --- | --- | --- | --- | --- | --- | --- | --- | --- |
| \| | # | Beta | \| | 9 | ( | 20%) | \| | - \| |
| \| | # | Coil | \| | 26 | ( | 57%) | \| | - \| |

|------------------------------|---------------------|---------------------|

| # Turn | 8 ( 17%) | - |

|------------------------------|---------------------|---------------------|

HYDROGEN BONDS (hbonds)

|------------------------------|---------------------|---------------------|

| Statistic | Observed | Expected |

|------------------------------|---------------------|---------------------|

| \| | Mean hbond | distance | \| | 2.3 | sd=0.4 | \| | 2.2 | sd=0.4 | \| |
| --- | --- | --- | --- | --- | --- | --- | --- | --- | --- |
| \| | Mean hbond | energy | \| | -1.5 | sd=0.8 | \| | -2.0 | sd=0.8 | \| |
| \| | # res with | hbonds | \| | 28 | ( 62%) | \| | 33 | ( 75%) | \| |

|------------------------------|---------------------|---------------------|

Expected values obtained from Morris AL, MacArthur MW, Hutchinson EG and Thornton JM. Proteins. 1992 Apr;12(4):345-364.

DIHEDRAL ANGLES

|------------------------------|---------------------|---------------------|

| Statistic | Observed | Expected |

|------------------------------|---------------------|---------------------|

| \| | Mean Helix Phi | | \| | -71.8 | sd=10.5 | \| | -65.3 | sd=11.9 | \| |
| --- | --- | --- | --- | --- | --- | --- | --- | --- | --- |
| \| | Mean Helix Psi | | \| | -36.3 | sd=19.3 | \| | -39.4 | sd=25.5 | \| |
| \| | # res with Gauche+ Chi | | \| | 13 | ( 46%) | \| | 15 | ( 55%) | \| |
| \| | # res with Gauche- Chi | | \| | 3 | ( 10%) | \| | 5 | ( 20%) | \| |
| \| | # res with Trans Chi | | \| | 12 | ( 42%) | \| | 7 | ( 25%) | \| |
| \| | Mean Chi Gauche+ | | \| | -63.4 | sd=10.6 | \| | -66.7 | sd=15.0 | \| |
| \| | Mean Chi Gauche- | | \| | 67.2 | sd=27.9 | \| | 64.1 | sd=15.7 | \| |
| \| | Mean Chi Trans | | \| | 161.2 | sd=8.8 | \| | 168.6 | sd=16.8 | \| |
| \| | Std. | dev of chi pooled | \| | 11.65 | | \| | 15.70 | | \| |
| \| | Mean | Omega (\|omega\|>90) | \| | 178.5 sd=16.6 | | \| | 180.0 sd=5.8 | | \| |

| # res with |omega|<90 | 1 ( 2%) | - |

|------------------------------|---------------------|---------------------|

Expected values obtained from Morris AL, MacArthur MW, Hutchinson EG and Thornton JM. Proteins. 1992 Apr;12(4):345-364.

ACCESSIBLE SURFACE AREA (ASA)

|------------------------------|---------------------|---------------------|

| Statistic | Observed | Expected |

|------------------------------|---------------------|---------------------|

| \| | Total ASA | \| | 3200.7 | Angs**2 | \| | 2971.8 Angs**2 \| |
| --- | --- | --- | --- | --- | --- | --- |
| \| | ASA of backbone | \| | 475.8 | Angs**2 | \| | - \| |
| \| | ASA of sidechains | \| | 2724.9 | Angs**2 | \| | - \| |
| \| | ASA of C | \| | 2184.4 | Angs**2 | \| | - \| |
| \| | ASA of N | \| | 132.2 | Angs**2 | \| | - \| |
| \| | ASA of N+ | \| | 126.8 | Angs**2 | \| | - \| |
| \| | ASA of O | \| | 602.4 | Angs**2 | \| | - \| |
| \| | ASA of O- | \| | 94.9 | Angs**2 | \| | - \| |
| \| | ASA of S | \| | 60.0 | Angs**2 | \| | - \| |
| \| | Exposed nonpolar ASA | \| | 2126.3 | Angs**2 | \| | 1952.4 Angs**2 \| |
| \| | Exposed polar ASA | \| | 680.3 | Angs**2 | \| | 640.1 Angs**2 \| |
| \| | Exposed charged ASA | \| | 394.1 | Angs**2 | \| | 608.1 Angs**2 \| |
| \| | Side exposed nonpolar ASA | \| | 2095.7 | Angs**2 | \| | - \| |
| \| | Side exposed polar ASA | \| | 276.8 | Angs**2 | \| | - \| |
| \| | Side exposed charged ASA | \| | 352.4 | Angs**2 | \| | - \| |
| \| | Fraction nonpolar ASA | \| | 0.66 | | \| | 0.61 sd=0.03 \| |
| \| | Fraction polar ASA | \| | 0.21 | | \| | 0.20 sd=0.05 \| |
| \| | Fraction charged ASA | \| | 0.12 | | \| | 0.19 sd=0.05 \| |
| \| | Mean residue ASA | \| | 71.1 sd=48.1 | | \| | - \| |
| \| | Mean frac ASA | \| | 0.4 sd=0.3 | | \| | - \| |
| \| | % side ASA hydrophobic | \| | 22.87 | | \| | - \| |

|------------------------------|---------------------|---------------------|

Expected values obtained from Miller S, Janin J, Lesk AM and Chothia C. J Mol Biol. 1987 Aug 5;196(3):641-656..

ACCESSIBLE SURFACE AREA FOR EXTENDED CHAIN

|------------------------------|---------------------|---------------------|

| Statistic | Observed | Expected |

|------------------------------|---------------------|---------------------|

| \| | Extended | nonpolar ASA | \| | 4275.4 | Angs**2 | \| | - \| |
| --- | --- | --- | --- | --- | --- | --- | --- |
| \| | Extended | polar ASA | \| | 2043.8 | Angs**2 | \| | - \| |
| \| | Extended | charged ASA | \| | 733.1 | Angs**2 | \| | - \| |
| \| | Extended | side nonpolar ASA | \| | 4239.3 | Angs**2 | \| | - \| |
| \| | Extended | side polar ASA | \| | 445.3 | Angs**2 | \| | - \| |
| \| | Extended | side charged ASA | \| | 696.2 | Angs**2 | \| | - \| |

|------------------------------|---------------------|---------------------|

VOLUME

|------------------------------|---------------------|---------------------|

| Statistic | Observed | Expected |

|------------------------------|---------------------|---------------------|

| \| | Total volume (packing) | \| | 5033.7 Angs**3 | \| | 4842.9 Angs**3 | \| |
| --- | --- | --- | --- | --- | --- | --- |
| \| | Mean residue volume | \| | 111.9 sd=42.0 | \| | 125.0 sd=40.0 | \| |
| \| | Mean frac volume | \| | 1.0 sd=0.1 | \| | 1.0 sd=0.1 | \| |
| \| | Molecular weight | \| | 4473.27 | \| | - | \| |

|------------------------------|---------------------|---------------------|

Expected values obtained from Richards FM. Annu Rev Biophys Bioeng. 1977; 6:151-176.

********************************

* STEREO/PACKING QUALITY INDEX *

********************************

( 3 = best ) ( 0 = worst )

( * = indicates possible problem )

PRBLM...: * *** * ** * * * * **

| SEQUENCE: | MAGTGGTYGQ | PGHTGMAGTG | TLGTDGTGEK | KGIMDKIKEK | LPGQH | 45 |
| --- | --- | --- | --- | --- | --- | --- |
| TORSION.: | 3133332233 | 2322332323 | 3332232332 | 3323333333 | 33321 |  |
| OMEGA...: | 3302211101 | 2033333122 | 2010333303 | 3313303233 | 21200 |  |
| VDW.....: | 3333333333 | 3333333333 | 1233333333 | 3333333333 | 33333 |  |

------------------------------------------------------------- TOTAL...: 9768876667 7688998778 6575898968 9969969899 87854

***************************

* 3D PROFILE QUALITY INDEX *

****************************

( 9 = best ) ( 0 = worst )

( * = indicates possible problem )

| PRBLM...:  SEQUENCE: | ***  MAGTGGTYGQ | **  PGHTGMAGTG | TLGTDGTGEK KGIMDKIKEK | *  LPGQH | 45 |
| --- | --- | --- | --- | --- | --- |
| ENV.....: | 6655444555 | 5554456665 | 5667677777 6666665555 | 56545 |  |

|------------------------------|---------------------|---------------------|

| Statistic | Observed | Expected |

|------------------------------|---------------------|---------------------|

| \|  \| | Resolution  R Value | | | | \|  \| | | - \|  - \| | | | | - \|  - \| | | | |
| --- | --- | --- | --- | --- | --- | --- | --- | --- | --- | --- | --- | --- | --- | --- |
| \| | # | res | in | phipsi | core | \| | 30 | ( | 66%) | \| | 41 | ( | 90%) | \| |
| \| | # | res | in | phipsi | allowed | \| | 13 | ( | 28%) | \| | 3 | ( | 7%) | \| |
| \| | # | res | in | phipsi | generous | \| | 2 | ( | 4%) | \| | 0 | ( | 1%) | \| |
| \| | # | res | in | phipsi | outside | \| | 0 | ( | 0%) | \| | 0 | ( | 0%) | \| |
| \| | # | res | in | omega | core | \| | 19 | ( | 42%) | \| | 43 | ( | 96%) | \| |
| \| | # | res | in | omega | allowed | \| | 9 | ( | 20%) | \| | 1 | ( | 3%) | \| |
| \| | # | res | in | omega | generous | \| | 8 | ( | 17%) | \| | 0 | ( | 0%) | \| |
| \| | # | res | in | omega | outside | \| | 9 | ( | 20%) | \| | 0 | ( | 1%) | \| |
| \| | # packing defects | | | | | \| | 2 | | | \| | 3 | | | \| |
| \| | Free energy of folding | | | | | \| | -20.04 | | | \| | -28.53 | | | \| |
| \| | # res 95% buried | | | | | \| | 5 | | | \| | 3 | | | \| |
| \| | # buried charges | | | | | \| | 0 | | | \| | 0 | | | \| |

|------------------------------|---------------------|---------------------|

Expected values obtained from 1. Morris AL, MacArthur MW, Hutchinson EG and Thornton JM. Proteins. 1992 Apr;12(4):345-364. 2. Chiche L., Gregoret LM, Cohen FE and Kollman PA. Proc Natl Acad Sci U S A. 1990 Apr;87(8):3240-3243

***************

* END VADAR *

***************

********************************************************************************

- Package...: VADAR (c)
- Version...: 1.5, December 2007
- Location..: University of Alberta
- Protein Engineering Network of
- Centres of Excellence
- Input.....: /var/www/html/vadar/public_html/temp/1569487517/1569487517.txt_m0

* Date......: Thu Sep 26 08:45:18 2019

********************************************************************************

***********************************

* MAIN CHAIN INFORMATION PANEL *

***********************************

| RES. RES. SCND | HBOND | BTURN RES. FRAC. RES. | FRAC. PHI | PSI | OMEGA PRBLM |
| --- | --- | --- | --- | --- | --- |
| NUM. NAME STRUC | HBOND | BTURN ASA ASA VOL. | VOL. PHI | PSI | OMEGA PRBLM |

----------------------------------------------------------------------------------------

Chain A

| 1 | MET | CCC | C |  |  | 52.0 | 0.24 | 170.8 | 1.05 | 0.0 | 178.8 | -178.7 | P |
| --- | --- | --- | --- | --- | --- | --- | --- | --- | --- | --- | --- | --- | --- |
| 2 | ALA | CCC | C | 16A |  | 8.9 | 0.07 | 89.5 | 1.03 | 79.0 | -171.3 | 177.9 |  |
| 3 | GLY | BCB | B |  |  | 55.8 | 0.61 | 55.4 | 0.88 | 164.2 | -150.0 | 156.1 | O |
| 4 | THR | BCB | B | 14A |  | 107.8 | 0.71 | 104.2 | 0.89 | -68.5 | -29.0 | 169.8 | O |
| 5 | GLY | BCB | B | 14A |  | 8.8 | 0.10 | 68.8 | 1.09 | -145.8 | -138.7 | -168.1 | O |
| 6 | GLY | BCB | B | 13A |  | 0.0 | 0.00 | 73.6 | 1.17 | -174.5 | 63.0 | -163.4 | O |
| 7 | THR | BCB | B |  |  | 55.8 | 0.37 | 104.4 | 0.89 | -64.4 | 21.4 | -164.2 | O |
| 8 | TYR | CCB | C | 21A |  | 117.1 | 0.48 | 170.9 | 0.89 | -128.0 | 51.3 | 161.1 | O |
| 9 | GLY | CCC | C | 10A |  | 67.4 | 0.74 | 51.5 | 0.82 | 178.9 | -78.0 | 141.5 | O |
| 10 | GLN | CCC | C | 9A |  | 70.9 | 0.37 | 134.3 | 0.97 | -71.5 | 150.6 | 163.3 | O |
| 11 | PRO | BCC | C |  |  | 91.2 | 0.59 | 111.3 | 0.97 | -71.9 | -165.8 | 165.1 | O |
| 12 | GLY | BCC | C |  |  | 31.1 | 0.34 | 54.4 | 0.86 | 174.7 | -155.0 | 155.6 | O |
| 13 | HIS | BBB | B | 6A,23A |  | 107.9 | 0.53 | 134.1 | 0.86 | -51.7 | 160.0 | -177.4 |  |
| 14 | THR | BBB | B | 4A,5A |  | 116.4 | 0.77 | 102.9 | 0.88 | -53.6 | 117.3 | -175.3 |  |
| 15 | GLY | BBB | B |  |  | 12.2 | 0.13 | 55.0 | 0.87 | -143.5 | 155.4 | 176.1 |  |
| 16 | MET | BBB | B | 2A |  | 134.3 | 0.61 | 174.6 | 1.07 | -68.4 | 158.3 | 170.7 |  |
| 17 | ALA | CBC | C |  |  | 50.4 | 0.41 | 92.4 | 1.06 | -71.8 | -175.7 | -176.8 |  |
| 18 | GLY | CCC | C |  |  | 45.1 | 0.50 | 53.5 | 0.85 | 157.4 | -118.4 | -160.1 | O |
| 19 | THR | CCC | C |  |  | 134.1 | 0.89 | 99.9 | 0.86 | -145.0 | 5.6 | -169.9 | O |
| 20 | GLY | CCC | C |  |  | 28.9 | 0.32 | 60.4 | 0.96 | 138.8 | 44.2 | -166.7 | O |
| 21 | THR | CCB | C | 8A |  | 1.0 | 0.01 | 149.9 | 1.28 | -81.9 | 175.5 | -166.4 | V,O |
| 22 | LEU | CCB | C |  |  | 22.8 | 0.11 | 198.9 | 1.22 | -131.4 | 140.3 | -134.4 | V,O |
| 23 | GLY | CCB | C | 13A,25A |  | 2.9 | 0.03 | 63.1 | 1.00 | -132.9 | -38.6 | 164.8 | O |
| 24 | THR | CCC | C | 25A |  | 93.6 | 0.62 | 99.3 | 0.85 | -165.4 | -63.0 | 143.7 | O |
| 25 | ASP | CCC | C | 23A,24A |  | 85.3 | 0.54 | 105.3 | 0.92 | -86.1 | -152.3 | -174.1 |  |
| 26 | GLY | CCC | C |  |  | 27.3 | 0.30 | 60.7 | 0.96 | 82.8 | 13.0 | -170.6 |  |
| 27 | THR | CCC | C |  |  | 133.1 | 0.88 | 97.1 | 0.83 | -59.0 | 86.2 | -176.8 |  |
| 28 | GLY | CCC | C | 30A |  | 46.8 | 0.52 | 68.6 | 1.09 | -98.1 | -171.8 | 173.9 |  |
| 29 | GLU | CCC | C |  |  | 126.9 | 0.67 | 121.3 | 0.91 | -64.1 | -20.5 | -159.9 | O |
| 30 | LYS | CCC | C | 28A,33A | III | 92.7 | 0.43 | 155.6 | 1.01 | -70.2 | 81.5 | 173.9 |  |
| 31 | LYS | CHC | C | 35A | III | 137.2 | 0.64 | 156.8 | 1.02 | -47.3 | -37.2 | 171.8 |  |
| 32 | GLY | CHC | C | 36A | III | 33.0 | 0.36 | 55.1 | 0.88 | -47.2 | -54.1 | -176.3 |  |
| 33 | ILE | CHH | H | 30A,37A | III | 8.5 | 0.04 | 143.7 | 0.89 | -69.6 | -74.9 | -163.4 | O |
| 34 | MET | HHH | H | 38A |  | 98.9 | 0.45 | 175.0 | 1.07 | -78.1 | -26.1 | 178.2 |  |
| 35 | ASP | HHH | H | 31A,39A |  | 29.1 | 0.18 | 118.9 | 1.04 | -86.3 | -36.2 | 171.3 |  |
| 36 | LYS | HHH | H | 32A,40A |  | 111.4 | 0.52 | 168.3 | 1.09 | -80.8 | -11.6 | 156.5 | O |
| 37 | ILE | HHH | H | 33A,41A |  | 4.9 | 0.02 | 150.5 | 0.93 | -68.0 | -53.2 | -176.6 |  |
| 38 | LYS | HHH | H | 34A,35A |  | 130.9 | 0.61 | 150.0 | 0.97 | -66.3 | -18.7 | 169.9 | O |
| 39 | GLU | HHH | H | 35A |  | 103.1 | 0.54 | 128.3 | 0.96 | -86.4 | -32.0 | 173.5 |  |
| 40 | LYS | HHH | H | 36A,43A | III | 49.1 | 0.23 | 154.3 | 1.00 | -65.8 | -50.6 | -171.8 |  |

| 41 | LEU | HHH | H | 37A,43A | III | 105.6 | 0.51 | 145.3 | 0.89 | -58.2 | -40.8 | 169.0 | O |
| --- | --- | --- | --- | --- | --- | --- | --- | --- | --- | --- | --- | --- | --- |
| 42 | PRO | CHH | H |  | III | 114.7 | 0.74 | 98.3 | 0.85 | -58.4 | -19.0 | -163.1 | O |
| 43 | GLY | CCH | C | 40A,41A | III | 46.6 | 0.51 | 54.8 | 0.87 | -167.2 | 75.7 | -167.4 | O |
| 44 | GLN | CCC | C |  |  | 109.9 | 0.58 | 118.1 | 0.85 | -76.8 | 62.2 | 153.3 | O |
| 45 | HIS | CCC | C |  |  | 189.3 | 0.93 | 134.5 | 0.86 | 38.5 | -180.0 | 0.0 | C |

----------------------------------------------------------------------------------------

Notes on PRBLM column:

A - indicates possible problem with fractional ASA (fASA > 1.0)

V - indicates possible problem with fractional volume (fV < 0.8 or fV > 1.2) P - indicates possible problem with Phi and Psi angles (Morris, et al, 1992) O - indicates possible problem with Omega angle (omega > 170 or omega < -170) C - indicates cis peptide bond (-20 < omega < 20)

********************************************************************************

- Package...: VADAR (c)
- Version...: 1.5, December 2007
- Location..: University of Alberta
- Protein Engineering Network of
- Centres of Excellence
- Input.....: /var/www/html/vadar/public_html/temp/1569487517/1569487517.txt_m0

* Date......: Thu Sep 26 08:45:18 2019

********************************************************************************

**********************************

* SIDE CHAIN INFO PANEL *

**********************************

RES. RES. SCND BTURN SIDE FRAC. SFE CHI1 ENV. DISULF S-S NUM. NAME STRUC BTURN SURF(ASA) SURF(ASA) SFE ANGLE CLASS BOND DIST

--------------------------------------------------------------------------------

Chain A

| 1 | MET | C |  | 41.1 | 0.23 | -2.4 | -61.7 | B1C |
| --- | --- | --- | --- | --- | --- | --- | --- | --- |
| 2 | ALA | C |  | 8.5 | 0.10 | -1.2 | 360.0 | P1C |
| 3 | GLY | B |  | 27.3 | 0.52 | -0.4 | 360.0 | E0B |
| 4 | THR | B |  | 87.0 | 0.77 | -0.8 | -161.9 | E0B |
| 5 | GLY | B |  | 7.2 | 0.14 | -0.7 | 360.0 | P1B |
| 6 | GLY | B |  | 0.0 | 0.00 | -0.8 | 360.0 | P1B |
| 7 | THR | B |  | 37.7 | 0.33 | -1.0 | -71.6 | P1B |
| 8 | TYR | C |  | 111.0 | 0.55 | -1.6 | -73.6 | P2C |
| 9 | GLY | C |  | 34.9 | 0.67 | -0.3 | 360.0 | E0C |
| 10 | GLN | C |  | 65.8 | 0.44 | -1.7 | -166.6 | P1C |
| 11 | PRO | C |  | 91.1 | 0.69 | -0.7 | 31.9 | P2C |
| 12 | GLY | C |  | 12.1 | 0.23 | -0.6 | 360.0 | P1C |
| 13 | HIS | B |  | 106.7 | 0.64 | -1.6 | -80.6 | P2B |
| 14 | THR | B |  | 113.4 | 1.00 | -0.4 | -167.5 | E0B |
| 15 | GLY | B |  | 3.0 | 0.06 | -0.8 | 360.0 | P1B |
| 16 | MET | B |  | 134.3 | 0.74 | -0.7 | -69.3 | P2B |
| 17 | ALA | C |  | 44.6 | 0.52 | -0.7 | 360.0 | P1C |
| 18 | GLY | C |  | 19.9 | 0.38 | -0.5 | 360.0 | E0C |
| 19 | THR | C |  | 105.2 | 0.93 | -0.1 | -69.2 | E0C |
| 20 | GLY | C |  | 25.8 | 0.49 | -0.4 | 360.0 | E0C |
| 21 | THR | C |  | 0.0 | 0.00 | -1.5 | -150.1 | P1C |
| 22 | LEU | C |  | 21.9 | 0.13 | -2.4 | -62.8 | B1C |
| 23 | GLY | C |  | 1.7 | 0.03 | -0.8 | 360.0 | P1C |
| 24 | THR | C |  | 65.0 | 0.57 | -0.5 | 166.7 | P2C |
| 25 | ASP | C |  | 82.8 | 0.70 | -0.6 | 71.5 | E0C |
| 26 | GLY | C |  | 20.5 | 0.39 | -0.5 | 360.0 | E0C |
| 27 | THR | C |  | 104.3 | 0.92 | -0.2 | -157.9 | E0C |
| 28 | GLY | C |  | 26.5 | 0.50 | -0.4 | 360.0 | E0C |
| 29 | GLU | C |  | 121.7 | 0.78 | 0.1 | 37.4 | E0C |
| 30 | LYS | C | III | 90.2 | 0.51 | -1.6 | -144.3 | P1C |
| 31 | LYS | C | III | 137.1 | 0.78 | -1.6 | -52.7 | E0C |
| 32 | GLY | C | III | 30.4 | 0.58 | -0.4 | 360.0 | E0C |
| 33 | ILE | H | III | 8.5 | 0.05 | -2.4 | -176.7 | B1H |
| 34 | MET | H |  | 98.9 | 0.55 | -1.4 | -160.2 | P2H |
| 35 | ASP | H |  | 28.8 | 0.24 | -0.9 | -49.8 | P1H |
| 36 | LYS | H |  | 108.5 | 0.61 | -1.9 | -54.3 | P2H |
| 37 | ILE | H |  | 4.9 | 0.03 | -2.5 | 154.7 | B1H |
| 38 | LYS | H |  | 124.2 | 0.70 | -2.0 | 92.8 | P2H |
| 39 | GLU | H |  | 95.7 | 0.61 | -1.1 | -159.9 | P2H |
| 40 | LYS | H | III | 49.1 | 0.28 | -1.9 | 167.5 | B1H |

| 41 | LEU | H | III | 84.2 | 0.50 | -1.4 | -70.3 | P2H |
| --- | --- | --- | --- | --- | --- | --- | --- | --- |
| 42 | PRO | H | III | 85.0 | 0.64 | -0.8 | -27.0 | P2H |
| 43 | GLY | C | III | 39.7 | 0.76 | -0.2 | 360.0 | E0C |
| 44 | GLN | C |  | 84.4 | 0.56 | -0.9 | -63.8 | P2C |
| 45 | HIS | C |  | 134.1 | 0.81 | -1.0 | -44.2 | E0C |


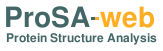


Please upload a structure in PDB format:

No file chosen


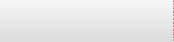


Choose File

[H_ELP_](https://prosa.services.came.sbg.ac.at/prosa_help.html)

Alternatively you can specify a structure by entering its PDB code, chain identifier and NMR model number:

PDB CODE: PDB CHAIN ID:

PDB MODEL NUMBER:

If you leave the fields for chain id or model number blank,

the first chain of the first model found in the PDB file will be analysed.


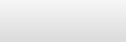


Analyse

**Results for model1.pdb, chain A (45 aa)**

# Overall model quality

*Z*-Score: **-1.76**

[H_ELP_](https://prosa.services.came.sbg.ac.at/prosa_help.html#output)


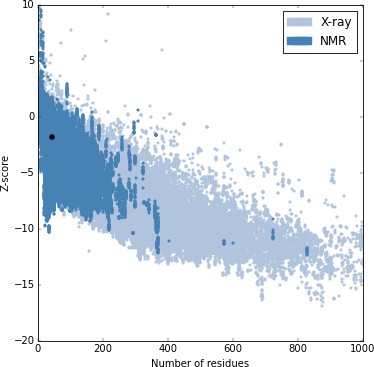


# Local model quality


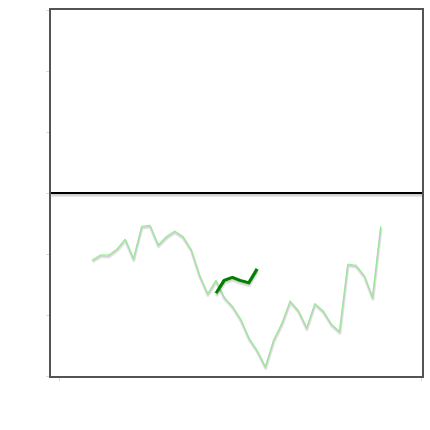

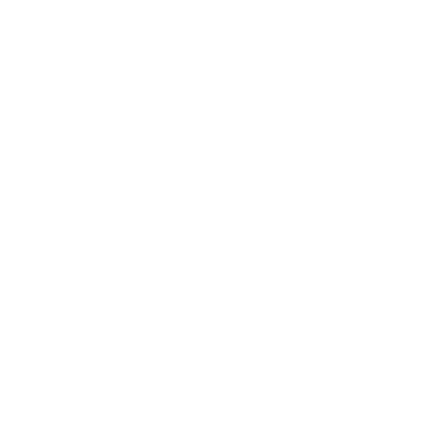


3.0

WINDOW SIZE 10

WINDOW SIZE 40

2.0

1.0

0.0

-1.0

-2.0

-3.0

1

45

Sequence position

Knowledge-based energy


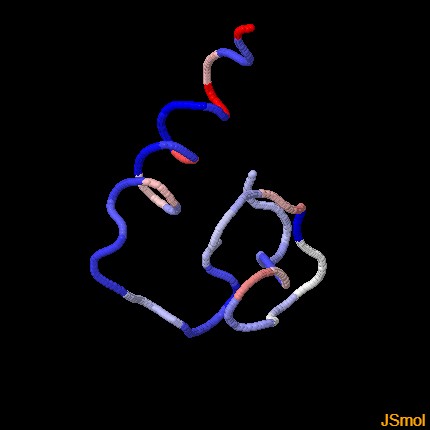
Lowest energy
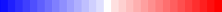
 Highest energy

[H_ELP_](https://prosa.services.came.sbg.ac.at/prosa_help.html#resplot)

Please cite the following articles if you publish results using ProSA-web:

Wiederstein & Sippl (2007)

ProSA-web: interactive web service for the recognition of errors in three-dimensional structures of proteins.

*Nucleic Acids Research* 35, W407-W410. [[view]](http://nar.oxfordjournals.org/cgi/content/short/35/suppl_2/W407) Sippl, M.J. (1993)

Recognition of Errors in Three-Dimensional Structures of Proteins.

*Proteins* 17, 355-362. [[view]](http://onlinelibrary.wiley.com/doi/10.1002/prot.340170404/abstract)

This site is maintained by Markus Wiederstein. For comments and suggestions please contact


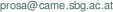
.
