# Supplementary material for: Data set of in-silico analysis and 3D modelling of boiling stable stress-responsive protein from drought tolerant wheat
Source: Data Brief. 2019 Oct 30;27:104657. doi: 10.1016/j.dib.2019.104657 (PMC6849113; doi:10.1016/j.dib.2019.104657)

**Supplementary Fig 5**

EMBOSS explorer

**OUTPUT FILE** [.stdout](http://www.bioinformatics.nl/emboss-explorer/output/421547/.stdout)

Created pepwheel.1.png

**IMAGE FILE** [pepwheel.1.png](http://www.bioinformatics.nl/emboss-explorer/output/421547/pepwheel.1.png)


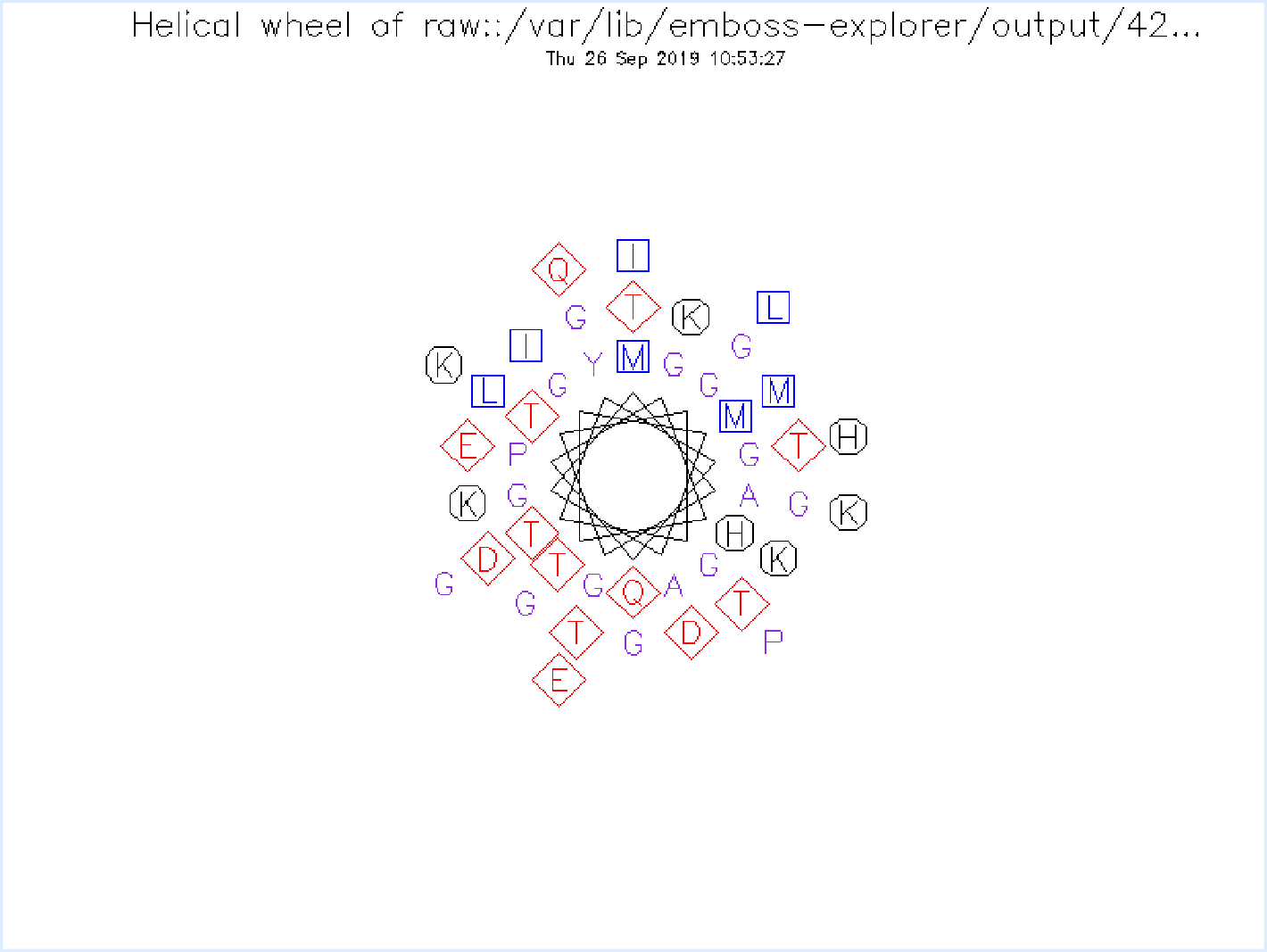

Supplement: Multimedia component 5 [file mmc5.docx]
